# Insights Into the Antibiofilm Activity of Carbon Quantum Dots Against a Panel of Different Microorganisms: A Review

**DOI:** 10.1002/mbo3.70411

**Published:** 2026-09-14

**Authors:** Hadeer M. Bedair, Mahmoud Hamed, Aly A. Shoun, Fotouh R. Mansour, Reem H. Obaydo, Tamer M. Samir

**Affiliations:** ^1^ Department of Microbiology and Immunology, Faculty of Pharmacy Misr University for Science and Technology Giza Egypt; ^2^ Pharmaceutical Chemistry Department, Faculty of Pharmacy Misr International University Cairo Egypt; ^3^ Microbiology and Immunology Department, Faculty of Pharmacy El‐Saleheya El‐Gadida University El‐Saleheya El‐Gadida Egypt; ^4^ Pharmaceutical Analytical Chemistry Department, Faculty of Pharmacy Tanta University Tanta Egypt; ^5^ Department of Analytical and Food Chemistry, Faculty of Pharmacy Ebla Private University Idlib Syria

**Keywords:** antimicrobials, biofilms, carbon quantum dots, review

## Abstract

Biofilm‐associated infections account for a majority of chronic bacterial diseases. These infections also present a therapeutic challenge due to their intrinsic tolerance to conventional antibiotics. Carbon dots (CDs) have emerged as a versatile nanoplatform with the potential to combat these resilient structures. This review critically evaluates the burgeoning field of CD‐based antibiofilm agents. We move beyond a catalog of studies to analyze how the physicochemical properties of CDs dictate their mechanisms of action against a panel of microorganisms. The antibiofilm activity of CDs is multi‐sided, stemming from their ability to (1) electrostatically interact with and disrupt the biofilm matrix and bacterial cell envelopes, (2) penetrate deep into biofilms due to their ultrasmall size, (3) generate ROS that degrade the extracellular polymeric substance (EPS) and kill embedded cells, and (4) downregulate key genes involved in quorum sensing and biofilm formation. An analysis of the literature reveals an efficacy bias towards Gram‐positive bacteria, highlighting the barrier posed by the outer membrane of Gram‐negative pathogens. We dissect how synthetic strategies and surface passivation techniques (e.g., with cationic polymers, lysozyme, or targeting ligands) are being deployed to overcome these challenges. While CDs represent a frontier in antibiofilm therapy, the field faces hurdles, including a lack of standardized testing protocols, limited in vivo validation, and unresolved questions about toxicity. This review identifies these critical gaps and proposes future research directions focused on the rational design of next‐generation CDs with enhanced specificity, potency, and translational potential for treating recalcitrant biofilm infections.

## Graphical abstract

Carbon quantum dots (CDs) have emerged as versatile nanoplatforms for combating biofilm‐associated infections through multiple mechanisms, including EPS disruption, membrane damage, ROS generation, and quorum sensing inhibition. This review highlights how CD physicochemical properties and surface engineering strategies influence antibiofilm activity against diverse microorganisms. Current challenges and future directions toward safe and clinically applicable CD‐based therapies are discussed.

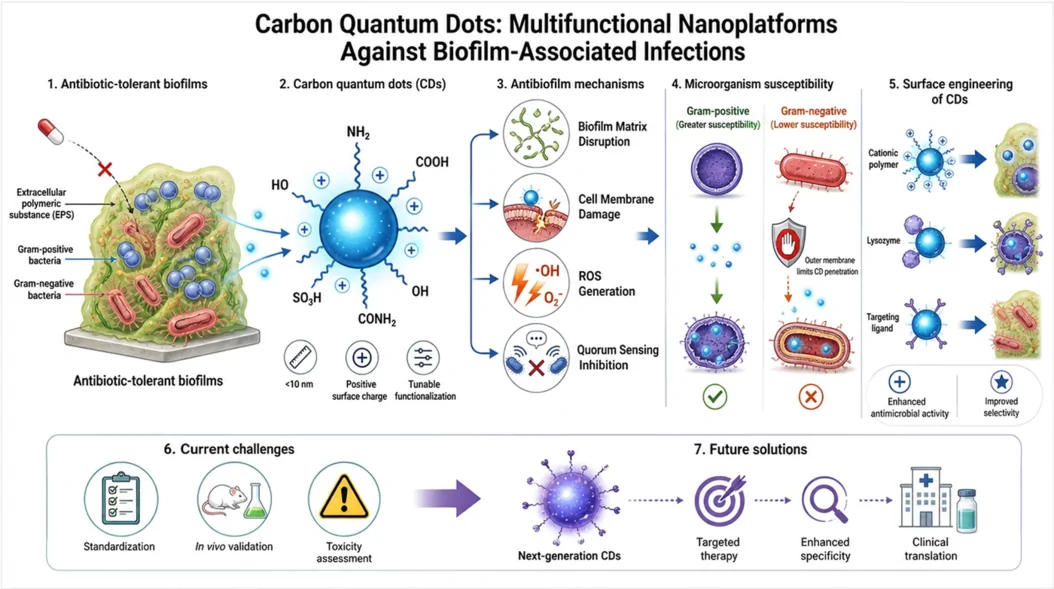

**Abbreviations**
BPs: bacteriophages
c‐di‐GMP: cyclic dinucleotide
CA: citric acid
Cur‐CDs: curcumin carbon dots
EPS: Extracellular Polymeric Substance
FA‐CDs: folic acid‐derived carbon dots
G‐CDs: guanidinium‐based carbon dots
LZM: lysozyme
MDR: multidrug‐resistant
MRAB: multidrug‐resistant *Acinetobacter baumannii*
MRST: multidrug‐resistant Salmonella Typhimurium
NMOFs: nanoscale metal‐organic frameworks
NPs: nanoparticles
NR: not reported
PEI: polyethyleneimine
PLA: Poly‐l‐lactic acid
PVAm: poly vinyl amine
QDs: quantum dots
QS: quorum sensing
ROS: reactive oxygen species
Si‐QAC: 3‐(trimethoxysilyl)‐propyldimethyloctadecyl ammonium chloride
S‐PCDs: spermidine‐capped carbon dots

## 1 Introduction

Bacterial biofilms are structured communities of cells entrenched in a self‐produced extracellular matrix of proteins, polysaccharides, and extracellular DNA (Vestby et al. 2020). This organized lifestyle represents one of the most prevalent modes of growth for pathogenic bacteria (Vani et al. 2023; Kalia et al. 2023), and it is estimated that 65%–80% of all infectious diseases are biofilm‐related (Lebeaux et al. 2013). These infections, which include chronic wounds, lung infections in cystic fibrosis patients, and device‐associated illnesses, are notoriously difficult to treat (Bjarnsholt 2013). A primary reason for this therapeutic failure is the remarkable tolerance of biofilm‐residing bacteria, which can withstand antibiotic concentrations up to 1000‐fold higher than their free‐swimming planktonic counterparts (Kalia et al. 2019). This intrinsic resistance, combined with enhanced tolerance to environmental stressors, renders conventional antibiotics largely ineffective and positions biofilms as a critical target for new therapeutic strategies (Kalia et al. 2023).

The formation of a biofilm is a dynamic, multi‐stage process involving initial bacterial attachment, maturation into a three‐dimensional community, and eventual dispersal of cells (Flemming et al. 2016). This process is tightly regulated by sophisticated signaling networks, including quorum sensing (QS) and the secondary messenger cyclic diguanylate (c‐di‐GMP), which control gene expression related to adhesion, matrix production, and virulence (Kalia et al. 2023, 2019). Consequently, modern antibiofilm strategies are shifting from simply killing bacteria to a more nuanced approach: inhibiting biofilm formation and disintegrating mature biofilms. This would re‐sensitize the exposed bacterial cells to antimicrobials at much lower, clinically achievable concentrations (Kalia et al. 2023).

To achieve this, a diverse arsenal of agents is being explored, including antimicrobial peptides, phytochemicals, enzymes, and bacteriophages (Kumar et al. 2021). Among these, nanoparticles (NPs) have garnered significant interest, not only for their intrinsic antimicrobial properties but also for their potential as targeted drug delivery carriers, which is a distinct advantage over the non‐specific distribution of traditional antibiotics (Karygianni et al. 2020). Within the broad class of NPs, quantum dots (QDs) have attracted considerable academic and industrial attention due to their size‐dependent optical properties (Kumar et al. 2021). However, conventional QDs often rely on expensive precursors and suffer from poor reproducibility, prompting a search for more economical, scalable, and eco‐friendly alternatives (Shaikh et al. 2018). This has led to the rise of CDs.

**Figure 1:**
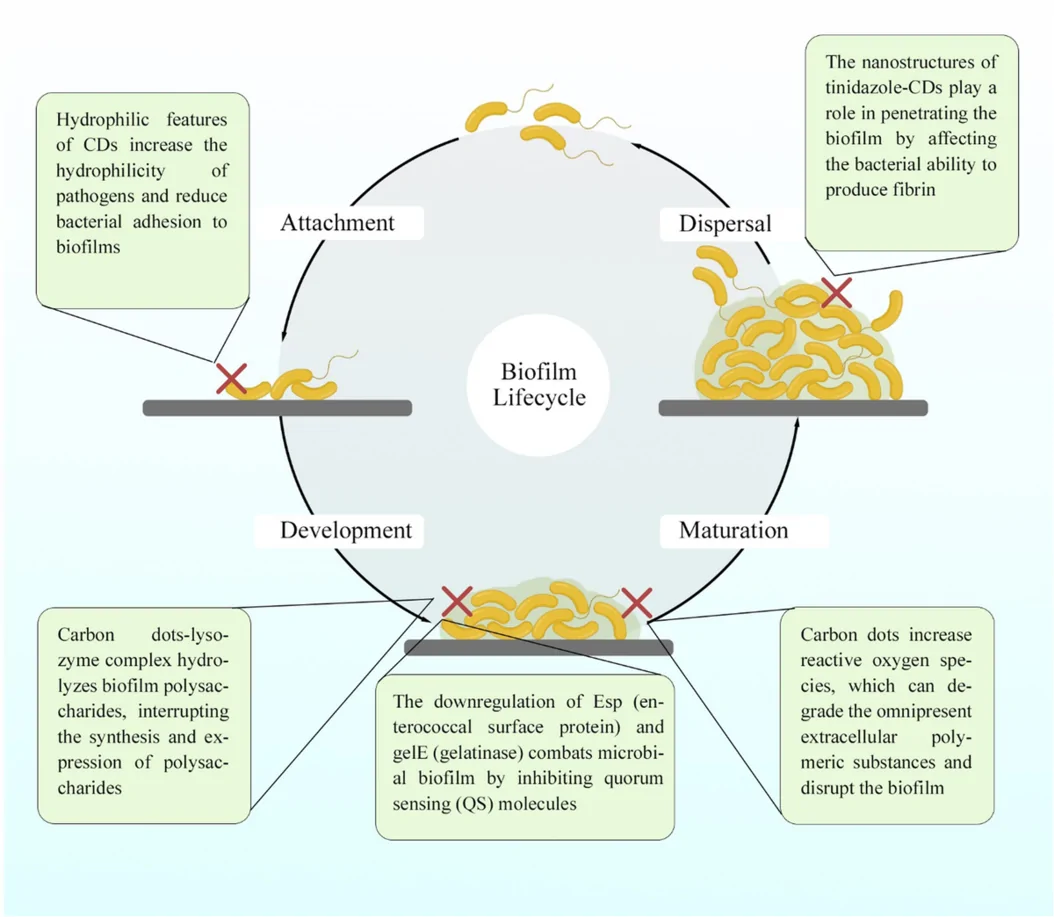
Biofilm lifecycle and potential modes of action of CDs against bacterial biofilm.

CDs are a new class of fluorescent carbon nanomaterials, typically below 10 nm in size, that have emerged as a uniquely tunable platform for biomedical applications (Shaikh et al. 2018). Their exceptional properties (including high photochemical stability, excellent biocompatibility, and low toxicity) have already led to their use in cellular imaging, biosensing, and drug delivery (Shaikh et al. 2018; Ran et al. 2019; Li et al. 2021, Li et al. 2020). Crucially, the synthetic flexibility of CDs allows for unparalleled control over their physicochemical characteristics. The choice of precursor and synthesis method is not merely a matter of preparation; it is a design strategy to intentionally imbue CDs with specific surface functionalities, such as amines for a positive charge, carboxylic acids for a negative charge, or hydrophobic domains. These properties directly govern their interaction with the complex biofilm matrix and resident microbial cells. Figure 1 provides a visual summary of the potential mechanisms by which CDs can disrupt the biofilm lifecycle.

## 2 Preparation and Characterization of CDs With Antibiofilm Activity

The preparation of CDs generally follows either a top‐down or bottom‐up approach. Among bottom‐up approaches, hydrothermal, solvothermal, microwave‐assisted, pyrolysis, and thermal decomposition are the most widely employed methods for CD synthesis. These methods convert small organic molecules or biomass precursors into nanosized carbon cores through dehydration, polymerization, aromatization, and carbonization processes. Compared with top‐down strategies, bottom‐up methods generally provide higher yields, better control over surface functional groups, and greater flexibility in precursor selection (Lamba et al. 2024). Microwave‐assisted synthesis has emerged as one of the most efficient bottom‐up approaches for preparing CDs because microwave irradiation heats the reaction mixture volumetrically through dielectric heating. Unlike conventional heating, where heat is transferred from the vessel walls to the solution, microwave irradiation enables rapid and relatively uniform heating throughout the reaction medium, significantly shortening reaction times from several hours to only a few minutes. This rapid heating promotes homogeneous nucleation, enhances carbonization efficiency, and often improves fluorescence properties and reaction yield (Ren et al. 2024). The method is compatible with a broad range of precursors, including citric acid, glucose, amino acids, polymers, and numerous biomass‐derived materials. In addition, nitrogen‐, sulfur‐, phosphorus‐, and boron‐doped CDs can be readily synthesized by incorporating suitable heteroatom‐containing precursors during microwave treatment. Because of its short reaction time, reduced energy consumption, and relatively simple operation, microwave‐assisted synthesis is considered an attractive strategy for sustainable and scalable CD production (Lamba et al. 2024). Despite these advantages, microwave‐assisted synthesis has several limitations. Product quality depends strongly on microwave power, reaction time, precursor composition, and solvent dielectric properties. Non‐uniform heating in domestic microwave ovens may result in broader particle size distributions and reduced reproducibility, while scale‐up remains challenging because microwave penetration depth decreases with increasing reaction volume. Therefore, dedicated laboratory microwave reactors are generally preferred for achieving reproducible and well‐controlled synthesis. The bottom‐up approach offers superior control over the final product's properties and is ideal for the green synthesis of CDs from a vast array of precursors (Khairol Anuar et al. 2021). These precursors range from biomass waste like fruit juices, milk, and honey to small molecules such as citric acid and polymers like polyethyleneimine (PEI) (Zhang, Li, et al. 2023; Jeong et al. 2023; Devkota et al. 2021; Cailotto et al. 2018; Pankaj et al. 2018; Cardoso‐Ávila et al. 2024; Mathew et al. 2020; Bhattacharyya et al. 2020; Oladzadabbasabadi et al. 2023; Atchudan et al. 2021; Preethi et al. 2022; Singh et al. 2020; Mandani et al. 2017; Gokul Eswaran et al. 2022; Chu et al. 2024; Vallan and Imahori 2022). In Table 1, a comparison of the principal CDs synthesis methods is presented. Information summarized and adapted from (Lamba et al. 2024; Ren et al. 2024; Ayisha Naziba et al. 2024).

**Table 1:** Advantages and limitations of CDs preparation methods.

Method	Advantages	Limitations
Hydrothermal	Simple, environmentally friendly, suitable for biomass precursors, and good surface functionalization	Long reaction time (4–24 h), high‐pressure autoclave required
Solvothermal	Better control over particle size and crystallinity, broader precursor compatibility	Organic solvents may increase toxicity and cost; longer reaction time
Microwave‐assisted	Very rapid (minutes), energy‐efficient, uniform volumetric heating, high yield, facile heteroatom doping	Difficult scale‐up, sensitive to microwave conditions, reproducibility may vary without dedicated reactors
Pyrolysis	Simple equipment, high productivity, suitable for bulk production	Higher temperatures, limited control of particle size and surface chemistry, and possible aggregation

For the purpose of designing effective antibiofilm agents, the choice of precursor is a critical design parameter. For instance, precursors rich in amines, such as PEI or spermidine, yield CDs with a cationic surface charge. This positive charge is strategically introduced to exploit the net negative charge of bacterial cell surfaces and the extracellular polymeric substance (EPS), thereby enhancing electrostatic binding and biofilm disruption (Ran et al. 2019; Sun et al. 2023). Conversely, precursors with abundant carboxyl groups, like citric acid, can produce negatively charged CDs, which may interfere with initial bacterial adhesion or target specific positively charged lipids in bacterial membranes (Cui et al. 2023; Agrawal et al. 2022). This tunability extends to the incorporation of hydrophobic segments, which can facilitate penetration through the EPS matrix, or specific targeting ligands, such as folic acid, to direct CDs to sites of infection (Yu et al. 2022, 2021).

**Figure 2:**
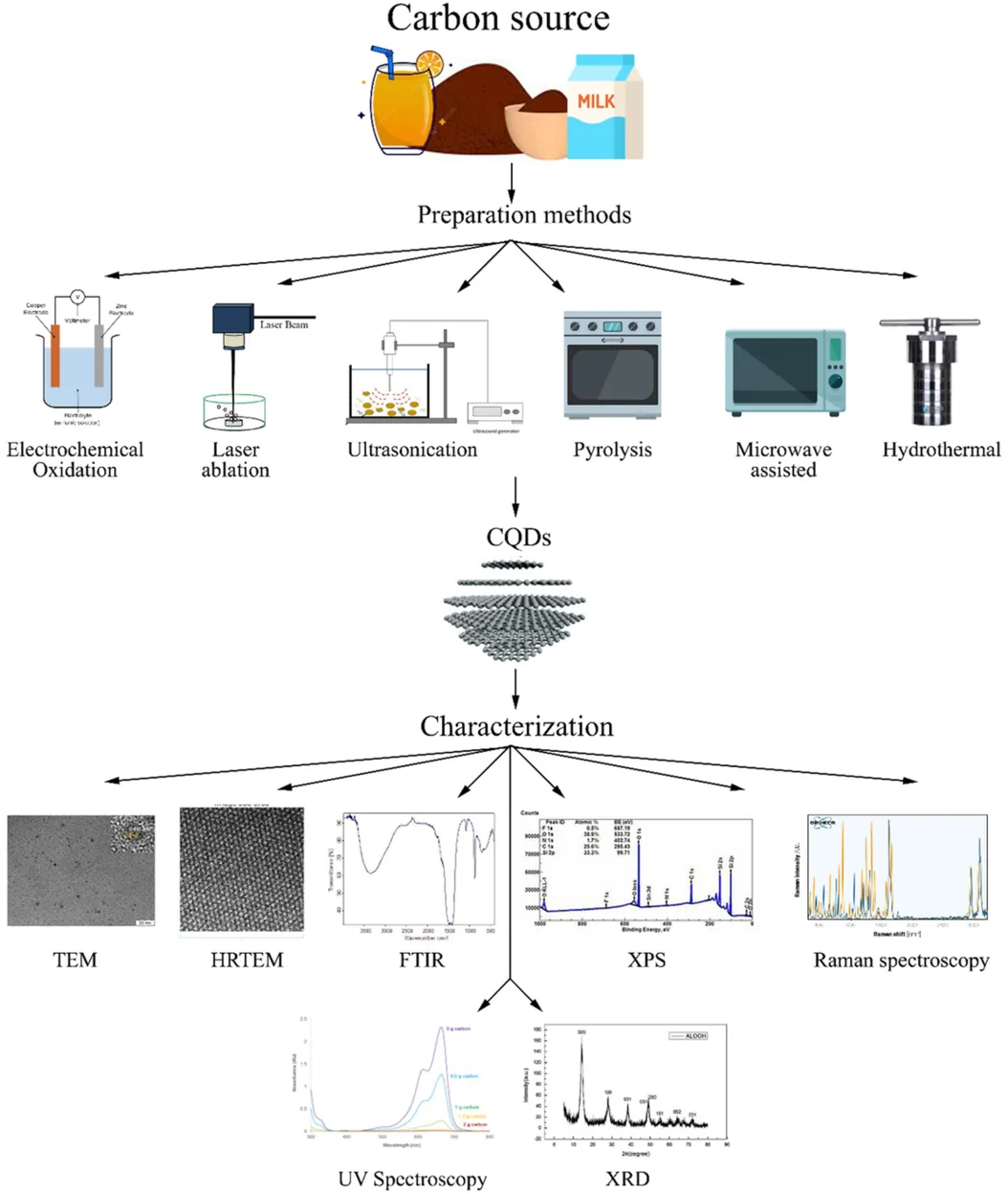
Methods of preparation and characterization of Carbon quantum dots.

Following synthesis, comprehensive characterization is essential, not as a routine checklist, but as the crucial link between a CD's designed properties and its subsequent biological activity. Transmission electron microscopy (TEM) confirms the ultrasmall size that enables deep biofilm penetration (Wang et al. 2020). Spectroscopic methods like Fourier‐transform infrared spectroscopy (FTIR) and x‐ray photoelectron spectroscopy (XPS) identify the precise functional groups imparted by the chosen precursor, verifying, for example, the successful introduction of amines (Wang et al. 2020; Hu et al. 2017). Crucially, zeta potential measurements provide a direct readout of surface charge, a parameter that will be repeatedly correlated with antibiofilm efficacy throughout this review. By integrating data from these techniques, we can begin to build a rational framework for understanding how specific CD features dictate their interactions with different microorganisms. This review will critically evaluate the recent advances in CD‐based antibiofilm agents, with a focus on how these engineered physicochemical properties dictate their mechanisms of action against Gram‐positive bacteria, Gram‐negative bacteria, and fungi, and will identify key challenges and future directions for translating this promising platform into the clinic. Figure 2 demonstrates the most common Methods of preparation and characterization of CDs.

## 3 Antibiofilm Activity of CDs Against Gram‐Positive Bacteria

The formidable barrier presented by the biofilm matrix, which severely limits drug penetration, renders Gram‐positive infections particularly recalcitrant to conventional therapy (Ran et al. 2019). In response, researchers have developed a diverse array of carbon dot (CD)‐based strategies, each designed to exploit specific vulnerabilities of Gram‐positive pathogens. This section critically analyzes these strategies, grouping them by their core mechanistic design to identify trends, compare efficacies, and highlight remaining challenges.

The most extensively explored approach relies on the intrinsic negative charge of the Gram‐positive cell wall, conferred by teichoic acids and the phospholipid membrane. By engineering CDs with a cationic surface charge, researchers aim to maximize electrostatic binding, leading to membrane disruption and cell death. Ran and colleagues developed metal‐free, quaternized CDs (Si‐QAC CDs) via solvothermal treatment of a quaternary ammonium silane and glycerol (Ran et al. 2019). Against *Staphylococcus aureus* (ATCC 6538), these CDs achieved 100% biofilm prevention at 1000 µg/mL, an effect attributed to the combined electrostatic and hydrophobic interactions of the quaternized surface with the bacterial envelope. This study elegantly demonstrated the core principle of cationic design, though the required concentration was relatively high.

Subsequent work has focused on intensifying this cationic charge and exploring its presentation. Abraham et al. (2022) used a hydrothermal process to create protonable PEI and citric acid‐based CDs. While effective against *S. aureus* biofilms, they required an even higher concentration of 1500 µg/mL, suggesting that the density or accessibility of the positive charges is critical. In contrast, Cui et al. developed spermidine‐capped CDs (S‐PCDs) that achieved 81.34% inhibition of *S. aureus* biofilms at just 16 µg/mL (Sun et al. 2023). This dramatically lower effective concentration highlights that not all cationic surfaces are equal; the polyamine structure of spermidine may provide a more optimized charge distribution or facilitate additional interactions.

The most potent example of this strategy to date comes from Zhang, Bai, et al. (2023), who developed guanidinium‐based CDs (G‐CDs). The guanidinium group is particularly interesting as it can form bidentate hydrogen bonds with negatively charged cell‐surface components, in addition to electrostatic attraction. At a concentration of only 20 µg/mL, G‐CDs reduced biofilm formation in methicillin‐resistant *S. aureus* (MRSA) to near‐zero levels (Zhang, Bai, et al. 2023). The stark contrast in efficacy between the PEI‐based CDs (1500 µg/mL), the quaternized CDs (1000 µg/mL), and the G‐CDs (20 µg/mL) highlights a critical point: *the specific molecular architecture of the cationic moiety is as important as the charge itself*. This variability, further exemplified by the 16 µg/mL efficacy of S‐PCDs, points to a need for systematic structure‐activity relationship studies to identify the optimal cationic motif for Gram‐positive targeting (Sun et al. 2023; Zhang, Bai, et al. 2023).

A second major strategy moves beyond simple electrostatic killing to incorporate “smart” features that enhance specificity and reduce off‐target effects. These designs aim to concentrate the therapeutic action at the infection site or release their payload only in response to the local bacterial environment. Yu and colleagues designed folic acid‐derived CDs (FA‐CDs) using a one‐step pyrolysis method (Yu et al. 2022). They hypothesized that residual folate groups on the CD surface would bind specifically to folate receptors, which are overexpressed on many bacterial species, including *S. aureus*. This targeting mechanism, combined with the ultrasmall size facilitating biofilm penetration, resulted in 82% disruption of mature *S. aureus* biofilms at 1000 µg/mL (Yu et al. 2022). Interestingly, the antibiofilm activity was dependent on the pyrolysis temperature, with FA‐CD ~ 280~ being most effective, illustrating how synthetic parameters can fine‐tune biological outcomes.

In a more complex example, Li et al. (2020) engineered a pH‐responsive dissociable nanosystem (PPD@CDLys). This system was designed to remain inert during transit but activated specifically within the acidic microenvironment of a growing biofilm. The self‐assembled NPs could penetrate the dense biofilm, where the acidic pH triggered hydrolysis, causing disassembly. This released both a biocidal cationic polymer and the l‐lysine‐derived CD cores (CDLys). The freed CDLys then generated intracellular reactive oxygen species (ROS), degrading the EPS and killing embedded bacteria. At 250 μg/mL, this system achieved a 60% inhibition rate, which increased to complete inhibition at 1000 μg/mL (Li et al. 2020). This work is a prime example of a multi‐mechanistic, “smart” system that combines targeted delivery with both physical and oxidative killing.

### 3.1 Combination and Enhancement Strategies

Another powerful approach involves integrating CDs with other active agents to create synergistic effects, combining the unique properties of CDs with the proven activity of enzymes, drugs, or antibiotics. Li et al. incorporated gentamicin sulfate and ammonium citrate‐derived carbon quantum dots (CQD ~ AG~ ) into an injectable, self‐healing hydrogel (Li et al. 2021). This composite (CQD ~ AG ~ ‐hydrogel) demonstrated 93.6% inhibition of *S. aureus* biofilm at 2 mg/mL. The key innovation was the acid‐labile Schiff base linkage, which allowed for the controlled release of the CQD ~ AG~ in response to the acidic environment created by metabolically active bacteria within the biofilm. This “on‐demand” release mechanism enhances efficacy while potentially minimizing systemic toxicity and drug resistance, a clear advantage over the free antibiotic gentamicin (Li et al. 2021).

A similar logic underpinned the work of Li et al. (2020), who directly calcined the antibiotic gentamicin sulfate to produce CQD ~ 180~. This process yielded CDs that retained the active structure of the parent antibiotic but gained additional antibacterial features, including a positively charged surface and the ability to induce ROS. The result was a highly potent agent that achieved > 99% destruction of *S. aureus* biofilms at a low concentration of 80 μg/mL, while also exhibiting low drug resistance (Li et al. 2020). This “conversion” strategy offers a compelling route to repurpose existing antibiotics into more potent, multi‐functional nanomaterials.

Zhao et al. (2023) explored a different type of synergy by coupling otherwise ineffective 4‐aminosalicylic acid‐derived CDs (4‐ACDs) with lysozyme (LZM) to form a CD‐LZM complex. While the 4‐ACDs alone showed no antibiofilm activity, the complex achieved an 86% inhibition rate against *S. aureus* biofilms at 60 μg/mL. The mechanism here is complementary: the CDs likely facilitate delivery and penetration, while the LZM actively hydrolyzes the peptidoglycan components of the biofilm matrix. However, as the authors note, this complex inherited LZM's Gram‐positive specificity and was ineffective against Gram‐negative biofilms (Zhao et al. 2023). This limitation explicitly defines a key research gap: while enzyme‐CD conjugates are highly promising for Gram‐positive infections, different design strategies (such as the inclusion of outer membrane‐permeabilizing agents) are required to tackle the more complex Gram‐negative cell envelope.

### 3.2 Natural Product‐Derived and Other Strategies

In a move towards “greener” synthesis, Lu et al. (2021) developed water‐soluble CDs from curcumin and citric acid. At 500 μg/mL, these Cur‐CDs exhibited efficient antibiofilm activity against *S. aureus*. This study supports the exploration of natural products as a sustainable source of functional CDs, though their efficacy was moderate compared to some of the more potent engineered variants.

Beyond the common pathogen *S. aureus*, CDs have also shown promise against other Gram‐positive models, including spore‐formers. Dong et al. (2021) used photoactive EDA‐CDs to achieve 100% inhibition of *Bacillus subtilis* biofilm development at 30 μg/mL under visible light. Similarly, Sutekin et al. demonstrated that N‐doped CDs could totally inhibit *B. subtilis* biofilm at 25 mg/mL, noting a stronger effect against Gram‐positive than Gram‐negative bacteria, which they attributed to enhanced electrostatic interaction with the Gram‐positive cell envelope (Sutekin et al. 2021). The enormous difference in effective concentrations between these two studies (30 vs. 25,000 μg/mL) again highlights the profound impact of CD design and the lack of standardized testing, making direct comparisons across studies challenging. This variability is a critical point that the field must address to accelerate the translation of these promising materials.

## 4 Antibiofilm Activity of CDs Against Gram Negative Bacteria

Targeting Gram‐negative pathogens presents a substantially greater challenge than their Gram‐positive counterparts. This inherent difficulty stems from the presence of an additional outer membrane, a complex asymmetric bilayer rich in lipopolysaccharide (LPS), which functions as a formidable permeability barrier against antimicrobial agents (Sutekin et al. 2021). Consequently, the design strategies for CDs must be more sophisticated to breach this protective layer. This section critically evaluates the various approaches researchers have employed to engineer CDs capable of overcoming the Gram‐negative outer membrane and disrupting biofilms.

**Figure 3:**
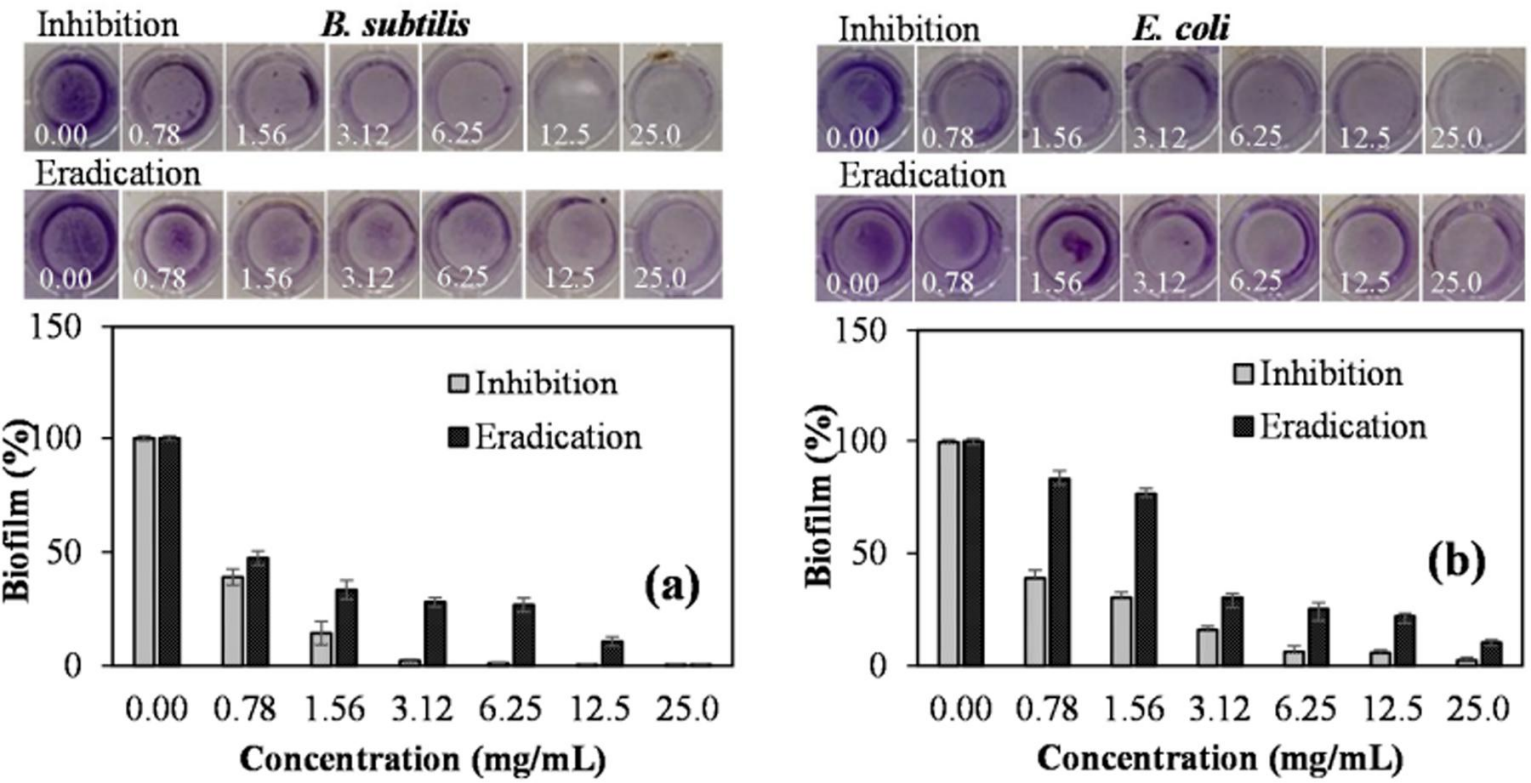
The photographs and prevention and eradication% of biofilms generated by (a) *Bacillus subtilis* and (b) *Escherichia coli* treated with N‐doped CDs synthesized by CA: PVAm at a 1:3 weight ratio using the crystal violet assay. Reproduced with permission from Sutekin et al. (2021).

A clear illustration of this challenge comes from the work of Sutekin et al. who developed nitrogen‐doped CDs (N‐doped C‐dots) via a hydrothermal method (Sutekin et al. 2021). While these cationic CDs achieved 100% biofilm inhibition against *B. subtilis* at 25 mg/mL, they required the same high concentration to inhibit *Escherichia coli* ATCC 8739. The authors correctly attribute this disparity to the thicker, more complex LPS layer of *E. coli*, which attenuates the electrostatic interaction that is so effective against Gram‐positive cells (Sutekin et al. 2021). This study provides a crucial baseline: simple cationic charge, while necessary, is often insufficient for potent Gram‐negative activity, demanding more elaborate design solutions. Figure 3 illustrates this differential activity directly, showing the inhibition of *B. subtilis* and *E. coli* biofilms treated with N‐doped CDs synthesized from citric acid and poly(vinyl amine) (PVAm) (Sutekin et al. 2021).

One approach to overcome the LPS barrier is to amplify the cationic charge or combine it with other bioactive components. Wang et al. adopted a “green” strategy, developing nitrogen and sulfur self‐doped garlic CDs (GCDs) via a one‐step hydrothermal method (Wang et al. 2023). Against a multidrug‐resistant *Salmonella Typhimurium* (MRST) strain, GCDs achieved 66.23% biofilm inhibition at 1600 μg/mL. The proposed mechanism is multifaceted: the cationic surface facilitates adsorption and membrane disruption, while the ultrasmall size enables internalization, leading to ROS accumulation and inhibition of antioxidant enzymes (Wang et al. 2023). Although effective, the concentration required is still relatively high, and the 66% inhibition rate against MRST leaves significant room for improvement, suggesting that while natural doping is a viable strategy, it may not yet rival the potency of more complex engineered systems.

A more powerful strategy involves integrating CDs into multi‐component nanocomposites, thereby introducing multiple, synergistic mechanisms of action to overwhelm the Gram‐negative cell envelope. Elkodous et al. engineered a sophisticated recyclable nanocomposite, Co_x_Ni_1_ − xFe_2_O_4_/SiO_2_/TiO_2_/C‐dots, using a layer‐by‐layer method (Abd Elkodous et al. 2020). This system combines the photocatalytic properties of TiO_2_ with the magnetic properties of the cobalt‐nickel ferrite core and the bioactivity of CDs. At a remarkably low concentration of just 15 µg/mL, it exhibited differential efficacy against a panel of Gram‐negative pathogens: 93.92% inhibition against *E. coli*, 50.35% against *Pseudomonas aeruginosa*, and 52.76% against *Klebsiella pneumoniae* (Abd Elkodous et al. 2020). The high efficacy against *E. coli* suggests that the photocatalytic generation of reactive species, combined with the physical properties of the nanocomposite, can effectively breach its outer membrane. However, the significantly lower activity against *P. aeruginosa* and *K. pneumoniae* highlights the variable defenses even among Gram‐negative species and highlights the need for pathogen‐specific optimization.

**Figure 4:**
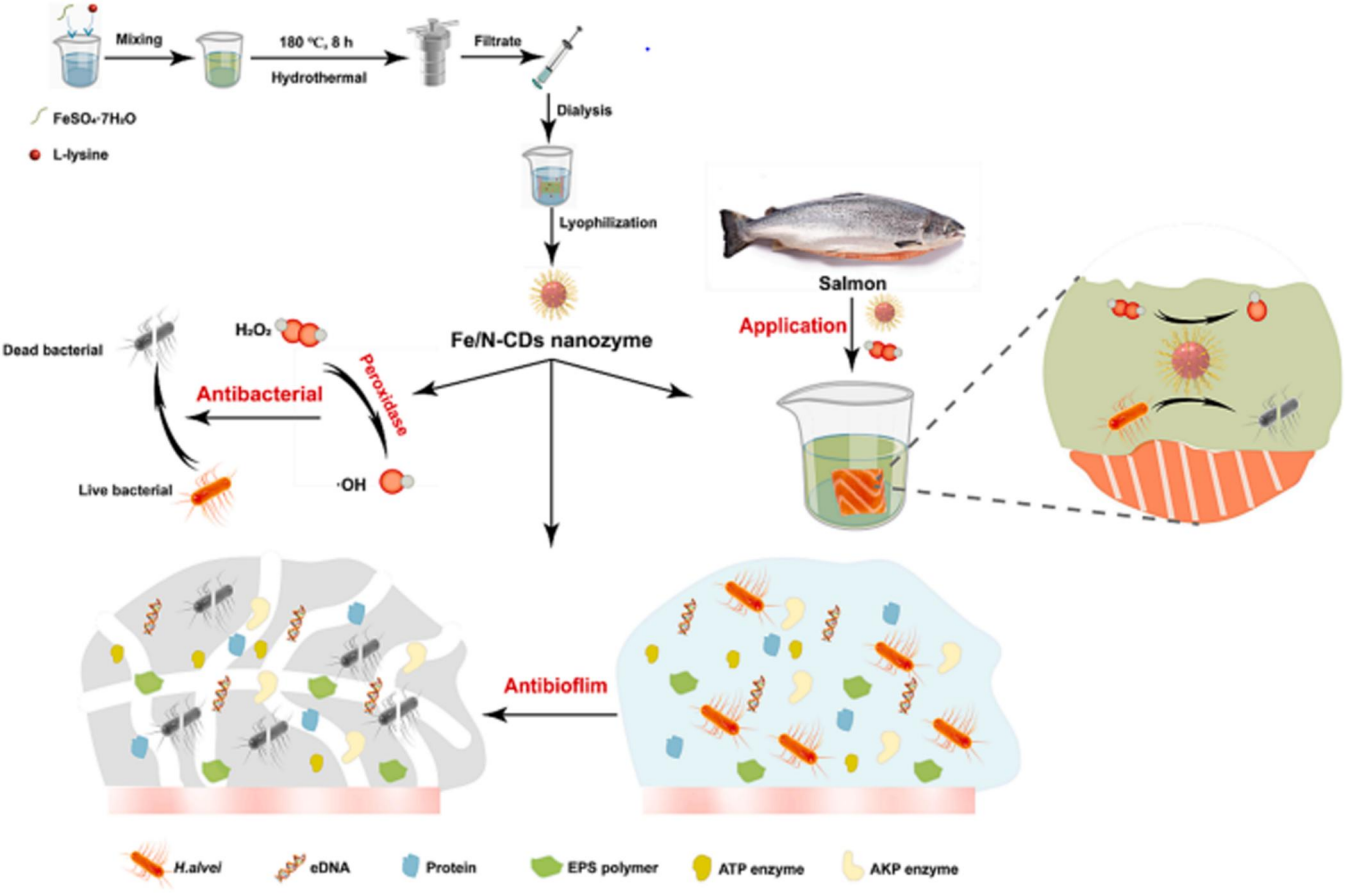
Synthesis processes, antibiofilm mechanism and application of the Fe/N‐CDs nanozyme. With the permission of Cui et al. (2023).

In a different approach, Cui et al. (2023) developed an Fe/N‐doped CD‐based nanozyme (Fe/N‐CDs) with potent peroxidase‐like activity. This nanozyme was tested against *Hafnia alvei*, a spoilage bacterium in aquatic products, achieving 73.2% biofilm inhibition at an exceptionally low concentration of 4 μg/mL. The mechanism here is catalytic: the Fe/N‐CDs mimic natural peroxidases, converting low concentrations of hydrogen peroxide into highly toxic hydroxyl radicals, which then degrade the EPS and kill embedded cells (Cui et al. 2023). Figure 4 provides a comprehensive visual summary of the synthesis, proposed antibiofilm mechanism, and food preservation application of this Fe/N‐CDs nanozyme (Cui et al. 2023). This nanozyme strategy is particularly noteworthy for two reasons. First, its effective concentration (4 μg/mL) is orders of magnitude lower than many other reported systems (as seen in Table 2), demonstrating the power of catalytic, rather than stoichiometric, modes of action. Second, it proved efficacious in a real‐world application, extending the shelf life of salmon by 3–6 days, showcasing the translational potential of well‐designed CD agents (Cui et al. 2023).

**Table 2:** Antibiofilm activity of carbon quantum dots against various microorganisms.

Tested microorganism	Strain	CD‐source	Method of preparation	Antibiofilm assay	Antibiofilm activity of CD	Microscopical study of biofilm	Ref.
*Bacillus cereus*	NR	Co_x_Ni_1−x_Fe_2_O_4_; *x* = 0.9/SiO_2_/TiO_2_/C‐dots)	Co‐precipitation (layer‐by‐layer)	Crystal violet staining	At 15 µg/ml biofilm inhibition 65.07%	SEM	Abd Elkodous et al. (2020)
*Bacillus subtilis*	ATCC 6633	N‐doped C‐dots (PVAm) and citric acid	Hydrothermal	Crystal violet staining	At 25 mg/mL 100% inhibition	NR	Sutekin et al. (2021)
*Bacillus. subtilis*	MTCC 121	Papaya leaf extracts	Hydrothermal	Standard pellicle (biofilm at the liquid–air interface) method	At its MIC and MIC/2	NR	Singh et al. (2021)
*Bacillus. subtilis*	NR	EDA‐CDs	Chemical functionalization	Crystal violet staining	At 30 µg/ml 100% biofilm inhibition.	NR	Dong et al. (2021)
*Candida albicans*	MTCC 227	Fruit juice of *Citrus limetta*	Solvothermal	MTT‐assay	40% reduction at 75 µg/mL	SEM	Shaikh et al. (2018)
*Candida albicans*	NR	Co_x_Ni_1−x_Fe_2_O_4_; *x* = 0.9/SiO_2_/TiO_2_/C‐dots)	co‐precipitation (layer‐by‐layer)	Crystal violet staining	At 15 µg/ml biofilm inhibition 66.29%	SEM	Abd Elkodous et al. (2020)
*Candida parapsilosis*	Clinical isolates (38)	urea and citric acid PLA/CDs	Solvothermal	Confocal microscopy and scanning electron microscopy	Biofilm inhibition 60% after 3 cycles treatment at 1.376 mm^2^) mats	CLSM and SEM	Mauro et al. (2020)
*Candida tropicalis*	NR	Co_x_Ni_1−x_Fe_2_O_4_; *x* = 0.9/SiO_2_/TiO_2_/C‐dots)	Co‐precipitation (layer‐by‐layer)	Crystal violet staining	At 15 µg/ml biofilm inhibition 92.35%	SEM	Abd Elkodous et al. (2020)
*Candida. albicans*	SC5314	CD‐Gu + ‐AmB	Hydrothermal	Colorimetric assay	At 100 μg/mL	CLSM, FEG and SEM	Li et al. (2019)
*Enterococcus. faecalis*	ATCC 29212	CDs derived from fucoidan	Hydrothermal	Live/dead fluorescence kit	At 3 mg/mL	CLSM	Tang et al. (2022)
*Escherichia coli*	ATCC 8739	N‐doped C‐dots (PVAm) and citric acid	Hydrothermal	Crystal violet staining	At 25 mg/mL 100% inhibition	NR	Sutekin et al. (2021)
*Escherichia coli*	LLC‐605	*Lactobacillus plantarum*	Hydrothermal	Crystal violet staining	At 3 mg/mL no biofilm formation	Confocal laser	Lin et al. (2018)
*Escherichia coli*		Artemisia argyi leaves	Smoking simulation method	live/dead staining assays		CLSM and SEM	
*Escherichia coli*	K12 MG1655	CDs‐C9	Hydrothermal	Crystal violet staining, live/dead staining assay, direct bright field microscopy	At 256 μg/mL, 70% bacteria death	TEM	Sviridova et al. (2022)
*Escherichia coli*	NR	Intravenous bags	Hydrothermal	CLSM	At 50 μg/mL −100 μg/mL	CLSM	Ramasubburayan et al. (2023)
*Escherichia coli*	ATCC25922	guanidinium‐based CDs (G‐CDs)	Solvothermal	Crystal violet staining, live/dead staining	At 20 μg/mL biofilm decreases to 43%	CLSM	Zhang, Bai, et al. (2023)
*Escherichia coli*	NR	Co_x_Ni_1−x_Fe_2_O_4_; *x* = 0.9/SiO_2_/TiO_2_/C‐dots)	Co‐precipitation (layer‐by‐layer)	Crystal violet staining	At 15 µg/mL biofilm inhibition 93.92%	SEM	Abd Elkodous et al. (2020)
*Escherichia coli*	ATCC 8739	Si‐QAC CDs	Solvothermal	Crystal violet staining, Live/dead staining assay and colony‐forming assay	At 1000 µg/mL biofilm biomass about 100%	Confocal	Ran et al. (2019)
*Escherichia coli*	NR	Curcumin carbon quantum dots Cur‐NRCQDs	Hydrothermal	Crystal violet staining	At residual biomass biofilm = 30%	Inverted fluorescence microscope	Su et al. (2022)
*Escherichia coli* and *Salmonella typhimurium*	ATCC 25922, ATCC 14028	*Bifidobacterium breve*	Hydrothermal	CLSM	26 to 14 µm^3^/µm^2^ reduction at 500 µg/mL	CLSM	Wu et al. (2021)
*Hafinia alvei*	NR	Fe/N‐CDs	Hydrothermal	Crystal violet staining	At 4 μg/mL inhibitory effect of biofilm 73.2%	SEM	Cui et al. (2023)
*Klebsiella pneumoniae*	NR	Co_x_Ni_1−x_Fe_2_O_4_; *x* = 0.9/SiO_2_/TiO_2_/C‐dots)	Co‐precipitation (layer‐by‐layer)	Crystal violet staining	At 15 µg/mL biofilm inhibition 52.76%	SEM	Abd Elkodous et al. (2020)
Methicillin resistant *Staphylococcus aureus*	ATCC43300	guanidinium‐based CDs (G‐CDs)	Solvothermal	Crystal violet staining, live/dead staining	At 20 μg/mL biofilm are almost zero.	CLSM	Zhang, Bai, et al. (2023)
Methicillin resistant S*taphylococcus aureus*	NR	Co_x_Ni_1−x_Fe_2_O_4_; *x* = 0.9/SiO_2_/TiO_2_/C‐dots)	Co‐precipitation (layer‐by‐layer)	Crystal violet staining	At 15 µg/mL biofilm inhibition 48.49%	SEM	Abd Elkodous et al. (2020)
Methicillin‐resistant *Staphylococcus aureus* (MRSA)	Multi drug resistant (MDR)	Nitrogen and sulfur self‐doped garlic CDs (GCDs	Hydrothermal	Crystal Violet Staining	At 1600 μg/mL the inhibitory effect = 91.78%	CLSM, SEM	Wang et al. (2023)
Multi drug‐resistant *Salmonella Typhimurium* (MRST)	Multi drug resistant (MDR)	Nitrogen and sulfur self‐doped garlic CDs (GCDs)	Hydrothermal	Crystal Violet Staining	1600 μg/mL the inhibitory effect = 66.23%	CLSM, SEM	Wang et al. (2023)
multidrug‐resistant *Acinetobacter baumannii*	Multidrug‐resistant	Red‐CD	Solvothermal	Crystal violet staining, live/dead staining	At 150 μg/mL relative biofilm biomass = 15%	CLSM	Liu et al. (2022)
*Porphyromonas gingivalis*	NR	Tinidazole carbon quantum dots and metronidazole carbon quantum dots	Hydrothermal	Crystal violet staining	At 100 μg/mL gene expression of biofilm formation regulation by *Fim A, Rgp A* reduced to 35 (relative expression)	CLSM	Liang et al. (2020)
*Pseudomonas aeruginosa*	ATCC 10145	Citric acid (CA) and cysteine (Cys)	Microwave assisted synthesis	Crystal Violet staining p	Biofilm was totally inhibited at 25 mg/mL	NR	Suner et al. (2021)
*Pseudomonas aeruginosa*	NR	Citric acid and curcumin	Hydrothermal	Crystal Violet staining	500 μg/mL	CLSM	Lu et al. (2021)
*Pseudomonas aeruginosa*	Clinical and control PAO1	Citrus medica fruit	Hydrothermal	Crystal Violet Staining	0.07% (v/v) reduced *P. aeruginosa* biofilm production 40%	Fluorescence Microscopy	Selvaraju et al. (2022)
*Pseudomonas aeruginosa*	Clinical isolates (31)	Urea and citric acid PLA/CDs,	Solvothermal	Confocal microscopy and scanning electron microscopy	Biofilm inhibition 80% after 3 cycles treatment at 1.376 mm^2^) mats	CLSM and SEM	Mauro et al. (2020)
*Pseudomonas aeruginosa*	NR	Co_x_Ni_1−x_Fe_2_O_4_; *x* = 0.9/SiO_2_/TiO_2_/C‐dots)	Co‐precipitation (layer‐by‐layer)	Crystal violet staining	At 15 µg/ml biofilm inhibition 50.35%	SEM	Abd Elkodous et al. (2020)
*Pseudomonas aeruginosa*	MTCC no. 741	Citrus aurantium Au@ OPCD_PEG_	Microwave	Crystal violet staining	At 300 µg/mL residual biomass biofilm 20%	CLSM	Sharma et al. (2021)
*Staphylococcus aureus*	ATCC 25923	Citric acid and branched polyethyleneimine (bPEI)	Hydrothermal	Crystal violet staining test, spread plate method	Reduction to 2% at 250 µg/mL of CQD2.5w	SEM, CLSM	Li, Yang, et al. (2020)
*Staphylococcus aureus*	ATCC 25923	CQD_AG2_‐hydrogel,	Hydrothermal	CLSM	93.6% Biofilm inhibition at 2 mg/mL	CLSM	Li et al. (2021)
*Staphylococcus aureus*	NR	Citric acid and curcumin	Hydrothermal	Crystal Violet staining	500 μg/mL	CLSM	Lu et al. (2021)
*Staphylococcus aureus*	ATCC 25923	PPD@ CDLy	Ultrasonic	Crystal violet staining	At 250 μg/mL inhibition rate is 60%. At 1000 μg/mL completely inhibit the biofilm.	SEM and CLSM	Li, Liu et al. (2020)
*Staphylococcus aureus*	NR	Quaternized carbon dot–papain complex (qCD–P)	Hydrothermal	Crystal violet staining	At 500 mg/ml destruction ratio = 90%	SEM and CLSM	Zhao et al. (2022)
*Staphylococcus aureus*	ATCC 25923	Folic acid derived CDs (FA‐CD)	Pyrolysis	Crystal violet staining	At 1000 μg/mL 82% antibiofilm	SEM and CLSM	Yu et al. (2022)
*Staphylococcus aureus*	NR	CDs‐LZM	Hydrothermal	The ameliorative crystal violet (CV) method	At 60 μg/mL the inhibition rate 86%	CLSM	Zhao et al. (2023)
*Staphylococcus aureus*	NR	Spermidine‐capped CDs (S‐PCDs)	Hydrothermal	Crystal violet staining	At 16 μg/mL Inhibition rate 81.34%	CLSM	Sun et al. (2023)
*Staphylococcus aureus*	ATCC25923	Modified Graphene quantum dots (m3GQDs)	Ultrasonicated‐assisted	Crystal violet staining	1:100 modified GQDs: 50%–60% biofilm degradation.	NR	Agrawal et al. (2022)
*Staphylococcus aureus*	ATCC 6538	(PEI) and citric acid (CA) based C‐dots	Hydrothermal	Crystal violet staining	At 1.5 mg/mL	NR	Abraham et al. (2022)
*Staphylococcus aureus*	ATCC® 43300™	CDs‐C9	Hydrothermal	Crystal violet staining, live/dead staining assay, direct bright field microscopy	At 64 μg/mL	TEM	Sviridova et al. (2022)
*Staphylococcus aureus*	Clinical isolates (62)	Urea and citric acid PLA/CDs	Solvothermal	Confocal microscopy and scanning electron microscopy	Biofilm inhibition 80% after 3 cycles treatment at 1.376 mm^2^) mats	CLSM and SEM	Mauro et al. (2020)
*Staphylococcus aureus*	NR	Intravenous bags	Hydrothermal	CLSM	At 50 μg/mL −100 μg/mL	CLSM	Ramasubburayan et al. (2023)
*Staphylococcus aureus*	ATCC6538	Guanidinium‐based CDs (G‐CDs)	Solvothermal	Crystal violet staining, live/dead staining	At 20 μg/mL biofilm are almost zero.	CLSM	Zhang, Bai, et al. (2023)
*Staphylococcus aureus*	ATCC 6538	Si‐QAC CDs	Solvothermal	Crystal violet staining, Live/dead staining assay and colony‐forming assay	At 1000 µg/mL biofilm biomass about zero.	Confocal	Ran et al. (2019)
*Staphylococcus aureus*	ATCC 25923	ZIF‐8@Lys‐CD@FA	Simple pyrolysis process	Crystal violet staining	At 500 µg/mL biofilm inhibition rate about = 70%	SEM and CLSM	Yu et al. (2021)
*Staphylococcus aureus*	NR	Curcumin carbon quantum dots Cur‐NRCQDs	Hydrothermal	Crystal violet staining	At 10 and 15 µM residual biomass biofilm = 30%	Inverted fluorescence microscope	Su et al. (2022)
*Staphylococcus aureus*	ATCC 25923	CQD_Gents_ (gentamicin)	Solvothermal followed by calcination.	Crystal violet staining	At 80 μg/mL 100% biofilm destruction at 180c calcination temp.	CLSM and SEM	Li et al. (2020)
*Streptococcus mutans*	NR	CHX‐CD	Hydrothermal	Crystal violet staining	NR	SEM and TEM	Ostadhossein et al. (2021)
Vancomycin‐resistant *Enterococcus. faecalis*	Clinical isolates	Curcumin CDs	Hydrothermal	Crystal violet staining	At 1000 μg/mL inhibition of biofilm 99.4%	NR	Sadaqat et al. (2022)

*Note:* NR: Not reported*.

**Figure 5:**
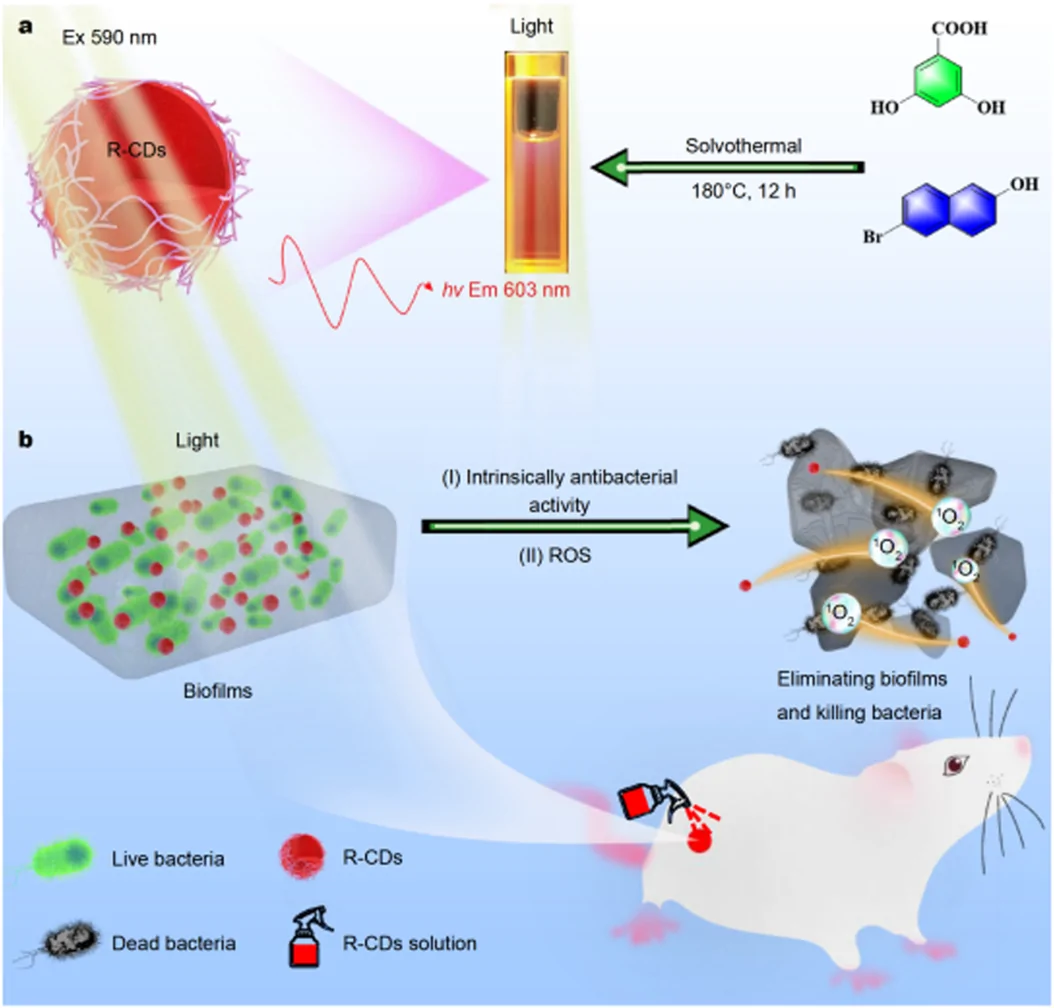
(a) Synthesis of R‐CDs by solvothermal processing. (b) Treatment of MRAB and its biofilms by photo‐excited R‐CDs. Reproduced with permission from Liu et al. (2022).

The rise of multidrug‐resistant Gram‐negative bacteria represents a critical global health crisis, and several studies have specifically targeted these formidable pathogens. The previously discussed GCDs from Wang et al. (2023) were effective against MDR *Salmonella*. Similarly, the TiO_2_‐based nanocomposite from Elkodous et al. was proposed for combating MDR bacteria, given its potent activity against a range of Gram‐negative species (Abd Elkodous et al. 2020). A particularly compelling example comes from Liu et al. (2022), who developed red‐emissive CDs (R‐CDs) via a solvothermal method using organic bactericide intermediates (2,4‐dihydroxybenzoic acid and 6‐bromo‐2‐naphthol) as precursors. These R‐CDs were designed for combined chemotherapeutic and photodynamic therapy. Against a multidrug‐resistant *Acinetobacter baumannii* (MRAB) strain, R‐CDs achieved 85% biofilm inhibition at 150 μg/mL under light activation (Liu et al. 2022). The photodynamic effect generates ROS upon light exposure, adding an additional, highly localized lethal mechanism to the CD's inherent activity. This design is particularly promising for localized infections, such as those on wounds or medical devices, where light activation can be applied externally. Figure 5 provides a schematic overview of this approach, illustrating both the synthesis of the R‐CDs and their proposed mechanism of action against MRAB biofilms under light activation (Liu et al. 2022).

### 4.1 CDs With Multifunctional Therapeutic Potential

Beyond direct antibiofilm activity, some CDs offer additional therapeutic benefits, hinting at their potential as multifunctional nanomedicines. Suner et al. developed cysteine‐derived CDs (Cys CDs) using a rapid microwave method (Suner et al. 2021). These S, N‐doped CDs completely inhibited *P. aeruginosa* ATCC 10145 biofilm growth at 25 mg/mL. Strikingly, the same Cys CDs also exhibited high antioxidant activity and significant inhibition of acetylcholinesterase (AChE), an enzyme implicated in Alzheimer's disease and diabetes‐related cognitive decline (Suner et al. 2021). While the concentration required for antibiofilm activity is high, this study opens an intriguing possibility: the development of multifunctional CDs that could simultaneously treat a biofilm‐associated infection and modulate a related neurological condition, though much work would be needed to reconcile the different effective concentrations for each application.

In summary, as collated in Table 2, the effective concentrations against Gram‐negative pathogens vary by over four orders of magnitude, from the 4 μg/mL of the Fe/N‐CDs nanozyme to the 25,000 μg/mL required for simple N‐doped or Cys‐CDs (Cui et al. 2023; Sutekin et al. 2021; Suner et al. 2021). This staggering range highlights the critical importance of rational CD design. The most potent systems appear to be those that incorporate multiple, synergistic mechanisms (such as photocatalytic activity (Abd Elkodous et al. 2020), nanozyme catalysis (Cui et al. 2023), or photodynamic therapy (Liu et al. 2022)) rather than relying on electrostatic interactions alone. Despite these advances, the mechanisms by which CDs traverse the formidable Gram‐negative outer membrane remain poorly understood. This highlights a critical research gap: future studies must combine efficacy testing with detailed investigations of CD uptake, intracellular trafficking, and the specific molecular interactions that enable these NPs to breach the LPS barrier. Furthermore, the promising activity against MDR strains (Wang et al. 2023; Abd Elkodous et al. 2020; Liu et al. 2022) suggests that CDs may offer a viable pathway to circumvent existing resistance mechanisms, a hypothesis that demands rigorous testing with panels of clinical MDR isolates.

## 5 Antibiofilm Activity of CDs Against Fungi

Fungal biofilms, particularly those formed by *Candida* species, represent a distinct and growing clinical challenge. Unlike bacterial biofilms, fungal biofilms are composed of a complex mixture of yeasts, hyphae, and pseudohyphae embedded in an extracellular matrix rich in β‐glucans and other polysaccharides (Shaikh et al. 2018). These structures are a major cause of morbidity in immunocompromised patients, leading to refractory infections associated with medical devices, mucosal surfaces, and deep‐seated tissues. The treatment of fungal biofilms is complicated not only by their intrinsic resistance to conventional antifungals but also by the fact that fungal cells are eukaryotic, making selective toxicity (killing the pathogen without harming host cells) substantially more difficult to achieve. This section critically evaluates the emerging but still limited body of research on CD‐based strategies against fungal biofilms.

**Figure 6:**
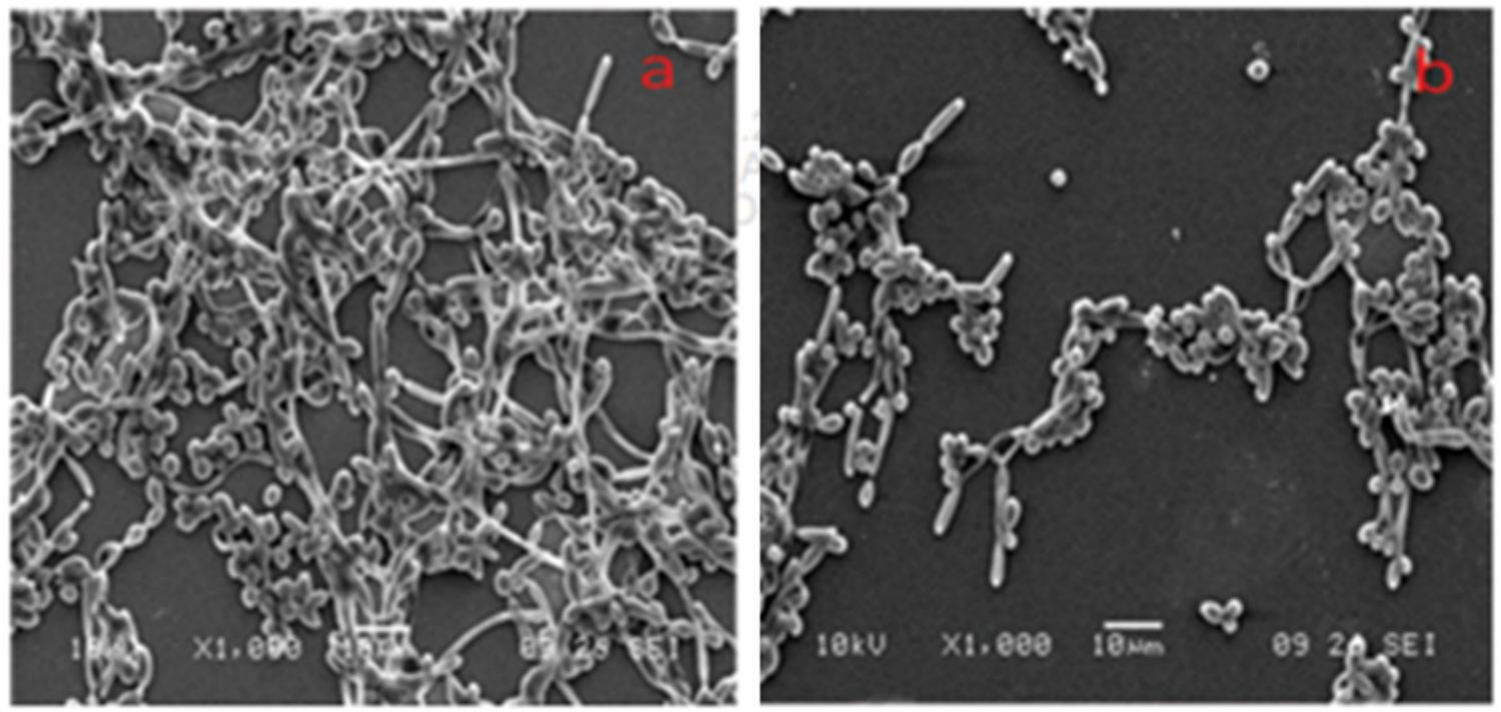
(a) Mycelia growth (biofilm) of *Candida albicans* as control group and (b) biofilm inhibition by the action of CDs. Reproduced with permission from Shaikh et al. (2019), Shaikh et al. (2018).

The simplest approach to developing antifungal CDs involves the use of natural precursors. Shaikh et al. synthesized colloidal luminescent CDs from *Citrus limetta* fruit juice using a solvothermal method and tested them against *Candida albicans* MTCC 227 (Shaikh et al. 2018). At a concentration of 75 µg/mL, these CDs achieved a 40% reduction in biofilm formation. Figure 6 visually confirms this antibiofilm effect, showing a clear reduction in *C. albicans* mycelial growth upon treatment (Shaikh et al. 2018).

The authors proposed two complementary mechanisms: inhibition of QS‐related processes and disruption of initial adhesion through electrostatic interactions between the CDs and the fungal cell wall, which interferes with attachment to the polystyrene surface (Shaikh et al. 2018). The abundance of hydroxyl groups on these CDs also presents an advantage for future functionalization in bioimaging and biomedical applications (Shaikh et al. 2018). However, the modest 40% inhibition rate at a relatively high concentration (75 µg/mL) establishes a clear baseline: simple, unmodified CDs from natural sources, while demonstrating proof‐of‐concept, may lack the potency required for clinical translation against fungal biofilms.

**Figure 7:**
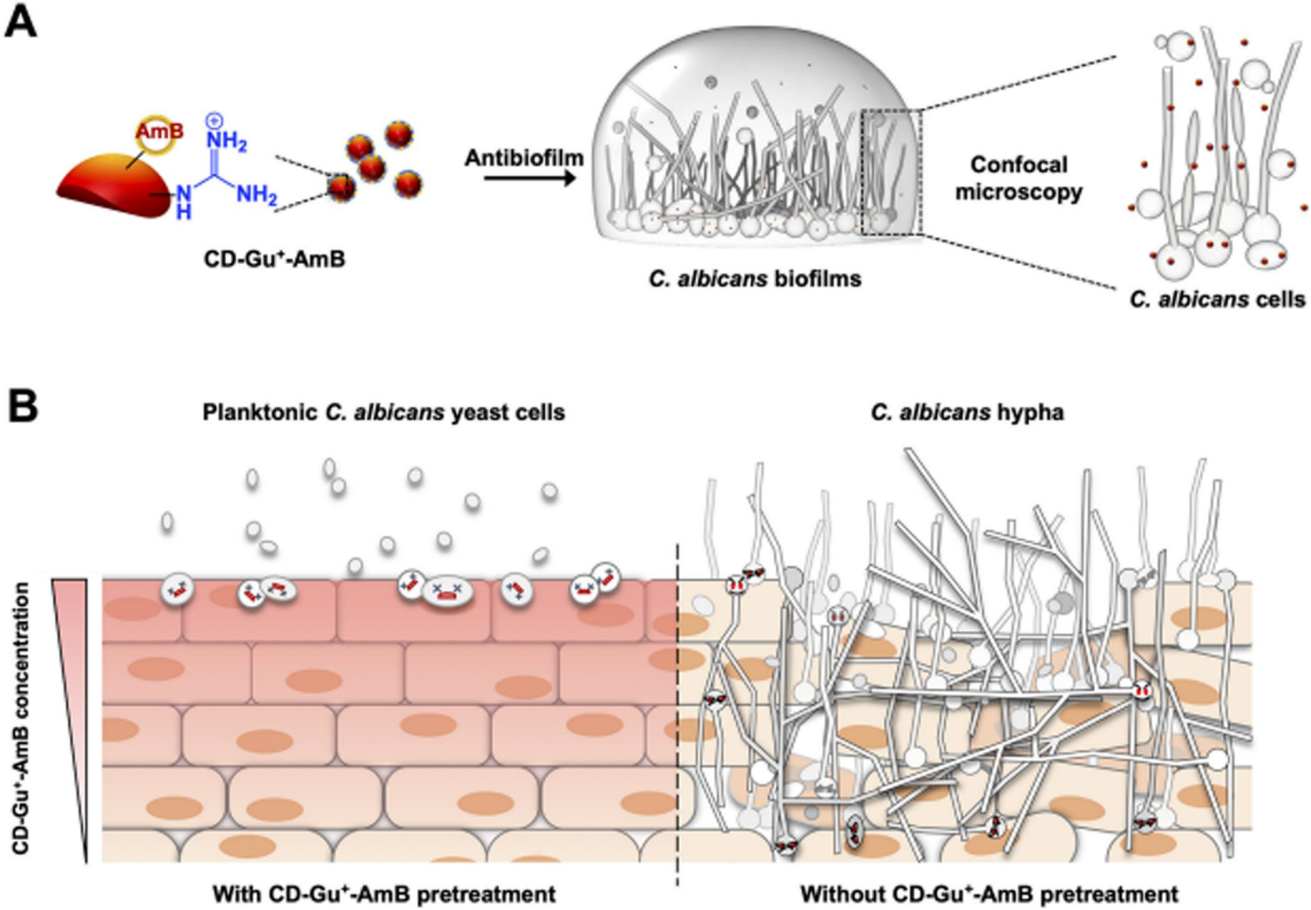
(A) Synthesized red‐emitting guanylated CDs with amphotericin B‐functionalization (CD‐Gu^+^‐AmB) present excellent antibiofilm effect on two‐day‐old biofilms of *Candida albicans*. Confocal microscopy shows accumulation in *Candida* cells leading to disrupted membrane integrity. (B) Pretreatment of CD‐Gu^+^‐AmB on Reconstituted Human Oral Epithelia (RHOE) ensures tissue integrity and protects against *C. albicans*. Reproduced with permission from Li et al. (2019).

A more sophisticated approach involves the rational design of CDs with specific antifungal moieties. Li et al. developed red‐emissive CDs featuring positively charged guanidine groups (Gu^+^) and subsequently conjugated them with the potent but toxic polyene antifungal amphotericin B (AmB) in a site‐controllable manner (Li et al. 2019). This design elegantly addresses multiple challenges simultaneously: the guanidine groups enhance electrostatic binding to the fungal cell surface, the red emission enables imaging, and the AmB provides potent, specific antifungal activity. At 100 µg/mL, the CD‐Gu^+^‐AmB conjugate significantly reduced biofilm formation in *C. albicans* SC5314 (Li et al. 2019). Figure 7 illustrates both the mechanism of action and a key safety assessment of this system. Panel A shows the accumulation of the conjugate in *Candida* cells and the resulting disruption of membrane integrity. Critically, Panel B demonstrates that pretreatment of Reconstituted Human Oral Epithelia (RHOE) with CD‐Gu^+^‐AmB preserved tissue integrity and protected it from *C. albicans* invasion, providing essential evidence of selective toxicity (Li et al. 2019).

This study represents a significant advance, demonstrating that functionalization of CDs with established antifungals can yield potent, targeted, and potentially safer agents. The effective concentration (100 µg/mL) is comparable to the simple fruit juice‐derived CDs, but the mechanism is far more specific and the outcome more complete, highlighting the value of rational design.

Elkodous et al. evaluated their Co_x_Ni_1_ − xFe_2_O_4_/SiO_2_/TiO_2_/C‐dots nanocomposite against two pathogenic fungi, *Candida tropicalis* and *C. albicans* (Abd Elkodous et al. 2020). At a concentration of just 15 µg/mL, the nanocomposite achieved 92.35% inhibition of *Candida tropicalis* biofilm and 66.29% inhibition of *C. albicans* biofilm (Abd Elkodous et al. 2020). This differential efficacy against two closely related species is striking and raises important questions. The near‐complete inhibition of *Candida tropicalis* suggests that the photocatalytic generation of ROS from the TiO_2_ component, combined with the physical properties of the nanocomposite, can be highly effective. However, the significantly lower activity against *C. albicans* highlights the variability in cell wall composition and defense mechanisms even within the same genus, highlighting the need for testing against multiple clinical isolates rather than single reference strains.

**Figure 8:**
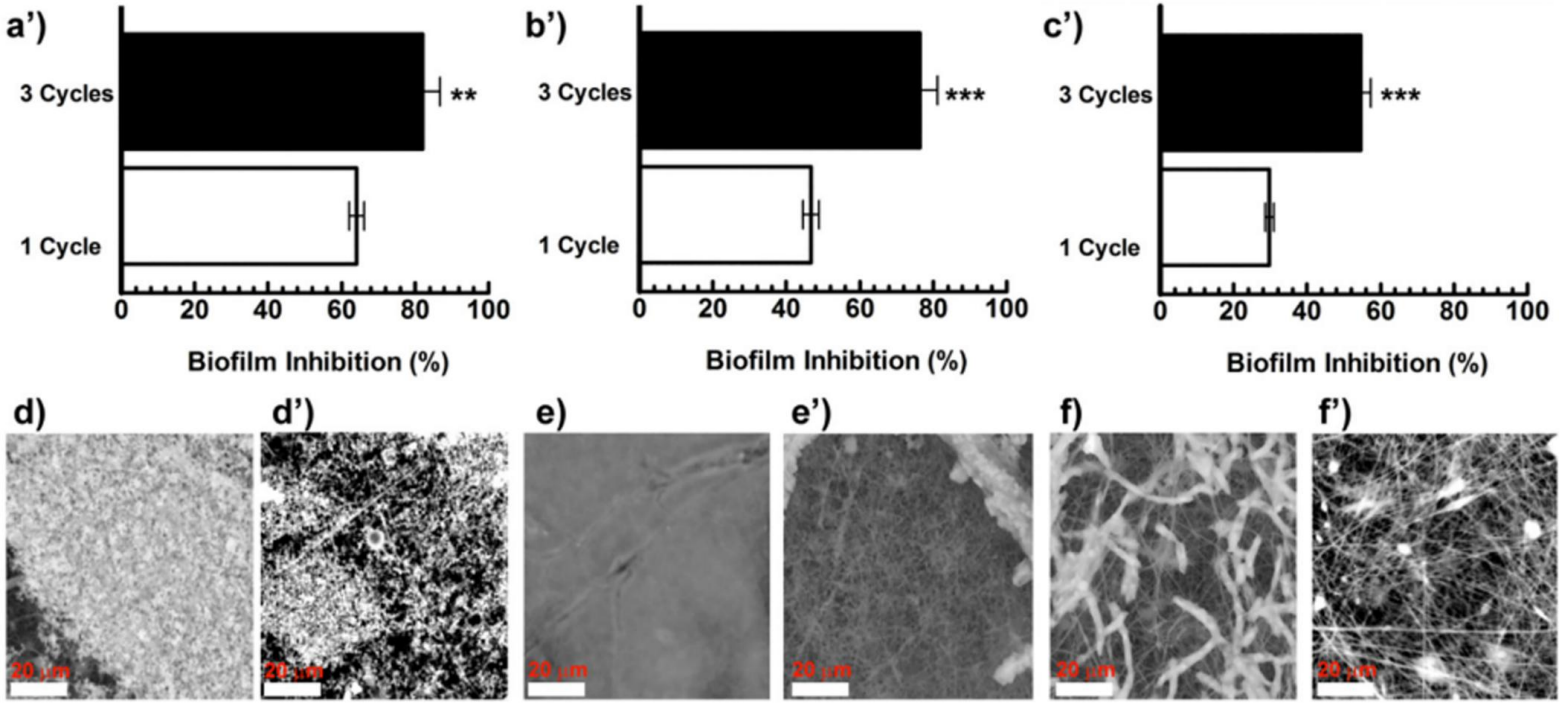
Biofilmicidal effectiveness against 24‐h‐old *Staphylococcus aureus* (a), *Pseudomonas aeruginosa* (b), and *Candida parapsilosis* (c) biofilms. Green‐stained microorganisms were imaged with 3D confocal microscopy Z‐stacks before and after NIR laser therapy (80 s, 5 W cm^−2^). Biofilm inhibition was measured by reducing fluorescence intensity in *S. aureus* 62 (a’), *P. aeruginosa* 31 (b’), and *Candida parapsilosis* 38 (c’) biofilms following 1 and 3 cycles of NIR laser treatment. Scanning electron microscopy of biofilms before (d–f) and after (d’–f’) treatment with NIR laser. Reproduced with permission from Mauro et al. (2020).

In a different but equally innovative approach, Mauro et al. engineered a scaffold composed of poly‐l‐lactic acid (PLA) doped with red‐emitting CDs using electrospinning (Mauro et al. 2020). This system was designed for photothermal therapy: the CDs efficiently convert near‐infrared (NIR) light into localized heat. Against clinical isolates of *Candida parapsilosis*, the PLA/CDs mats (1.376 mm^2^) achieved 60% biofilm inhibition upon NIR laser irradiation (Mauro et al. 2020). Figure 8 provides comprehensive visual evidence of this biofilmicidal effectiveness. Confocal microscopy Z‐stacks (a–c) show green‐stained microorganisms before and after NIR laser therapy. Quantification of fluorescence intensity (a’–c’) demonstrates significant biofilm reduction after one and three cycles of treatment. Scanning electron microscopy (d–f) reveals the structural integrity of 24‐h‐old biofilms before treatment, while the post‐treatment images (d’–f’) show clear disruption and cell death (Mauro et al. 2020).

The key advantage of this approach is its physical mechanism of action, which is unlikely to induce resistance. Furthermore, the PLA/CDs nanocomposite offers a practical pathway for creating self‐sterilizing medical devices, implants, and inserts that can be activated on demand to eradicate biofilm‐associated infections (Mauro et al. 2020).

Compared to the extensive literature on antibacterial CDs, studies targeting fungal biofilms remain relatively scarce. The available research, summarized in Table 2, reveals several trends and significant gaps. First, effective concentrations vary widely, from 15 µg/mL for complex nanocomposites (Abd Elkodous et al. 2020) to 100 µg/mL for functionalized systems (Li et al. 2019). Second, certain design features (specifically positive charge (guanidine groups) and the incorporation of known antifungals (AmB) or photothermal therapy) appear to enhance efficacy. Third, the marked difference in activity against different *Candida* species within the same study (Abd Elkodous et al. 2020) highlights the need for testing against diverse clinical isolates.

Critically, the mechanisms by which CDs exert selective toxicity against fungal cells without harming mammalian hosts remain poorly understood. While electrostatic interactions and ROS generation are frequently invoked, detailed investigations into fungal‐specific uptake, intracellular trafficking, and the molecular basis of membrane disruption are lacking. Future research must address these fundamental questions and expand the repertoire of fungal pathogens studied to include emerging drug‐resistant species such as *Candida auris*.

## 6 Interpretation of CDs Antibiofilm Modes of Action

The antibiofilm activity of CDs is not attributable to a single, universal mechanism but rather arises from a complex and often synergistic interplay of their physicochemical properties. The initial and most frequently cited interaction between CDs and their microbial targets is electrostatic. The bacterial cell surface is overwhelmingly negatively charged, due to teichoic acids in Gram‐positive bacteria and LPS in Gram‐negative species. The EPS matrix itself is also rich in anionic polysaccharides and extracellular DNA. Consequently, engineering CDs with a cationic surface charge is a logical and widely employed strategy to promote binding and subsequent disruption (Shaikh et al. 2018; Li et al. 2021, 2020; Yu et al. 2022, 2021; Abraham et al. 2022; Zhang, Bai, et al. 2023; Wang et al. 2023; Li, Yang, et al. 2020; Zhao et al. 2022; Sharma et al. 2021).

The work of Zhang et al. with guanidinium‐based CDs (G‐CDs) provides a powerful example (Zhang, Bai, et al. 2023). The strong positive zeta potential of these G‐CDs facilitates robust electrostatic interaction with the negatively charged bacterial membrane, leading to loss of membrane integrity and cell death. However, a critical comparison of cationic CD studies reveals that not all positive charges are created equal. The G‐CDs achieved near‐complete eradication of MRSA biofilms at just 20 μg/mL (Zhang, Bai, et al. 2023), while PEI‐based CDs required 1500 μg/mL for activity against *S. aureus* (Abraham et al. 2022), and quaternized Si‐QAC CDs needed 1000 μg/mL (Ran et al. 2019). This staggering range in efficacy, spanning nearly two orders of magnitude, highlights that charge density, the specific molecular presentation of the cationic moiety (e.g., guanidinium vs. primary amine vs. quaternary ammonium), and the three‐dimensional architecture of the CD surface are all critical determinants of electrostatic binding efficiency.

The limitations of this mechanism are most apparent when targeting Gram‐negative bacteria. The LPS layer of the outer membrane acts as a formidable barrier, attenuating electrostatic interactions that are highly effective against Gram‐positive cells (Sutekin et al. 2021; Zhao et al. 2022). As a result, many cationic CDs that perform well against *S. aureus* show significantly reduced efficacy against *E. coli* or *P. aeruginosa* (Sutekin et al. 2021). This Gram‐negative efficacy gap remains a major challenge, suggesting that simply maximizing positive charge is insufficient; strategies to actively facilitate outer membrane penetration are required.

Interestingly, some negatively charged CDs also exhibit antibiofilm activity through different mechanisms. For instance, Agrawal et al. demonstrated that negatively modified graphene quantum dots could interact with lysyl‐phosphatidyl glycerol, a positively charged phospholipid enriched in the *S. aureus* membrane, still enabling membrane disruption (Agrawal et al. 2022). Other studies suggest that negative charge may interfere with the initial adhesion of bacteria to surfaces rather than directly killing cells (Cui et al. 2023; Lin et al. 2018). This highlights that “antibiofilm activity” can be achieved through distinct pathways: bactericidal killing versus anti‐adhesion. Beyond pure electrostatics, hydrophobic interactions also play a role. CDs with amphiphilic properties, featuring a cationic head group attached to a hydrophobic alkyl chain, can insert into and physically disrupt the lipid bilayer, further compromising membrane integrity (Chen et al. 2020; Sviridova et al. 2022).

While surface binding is the first step, the antibiofilm activity of CDs is greatly enhanced by their ability to penetrate deep into the biofilm structure and, subsequently, into bacterial cells themselves. The ultrasmall size of CDs (typically < 10 nm) is a critical advantage. This nanoscale dimension allows CDs to diffuse through the water channels and porous architecture of the EPS matrix, which often excludes larger molecules and many conventional antibiotics. Numerous studies have used confocal microscopy to visually confirm the deep penetration of CDs into mature biofilms of *S. aureus* (Li et al. 2020; Yu et al. 2022; Abraham et al. 2022; Zhang, Bai, et al. 2023; Li, Yang, et al. 2020). *P. aeruginosa* (Liu et al. 2022; Mauro et al. 2020), and *C. albicans* (Sadaqat et al. 2022; Tang et al. 2022). This ability to reach the innermost layers of the biofilm is essential for eradicating the metabolically inactive “persister” cells that frequently repopulate the biofilm after treatment.

Once internalized by bacterial cells, a common downstream consequence is the induction of ROS. A substantial body of work has correlated antibiofilm efficacy with elevated intracellular ROS levels (Li et al. 2021, 2020; Cui et al. 2023; Yu et al. 2022, 2021; Zhang, Bai, et al. 2023; Abd Elkodous et al. 2020; Suner et al. 2021; Zhao et al. 2022; Su et al. 2022; Ramasubburayan et al. 2023). The proposed mechanism is that CDs, potentially through surface defects, quinone groups, or other photochemical properties, trigger an imbalance in the bacterial oxidative stress response, leading to oxidative damage to proteins, lipids, and DNA. In the context of biofilms, ROS serve a dual function: they kill embedded bacteria and simultaneously degrade the EPS matrix itself, breaking down the polysaccharides and eDNA that provide structural integrity (Wu et al. 2021). Wu et al. traced the source of this ROS generation to heterocyclic aromatic amines formed during the carbonization of their probiotic‐derived B‐C‐dots, providing a valuable insight into the structure‐activity relationship (Wu et al. 2021). The synergy between size‐dependent penetration and ROS generation is key. The ultrasmall size enables deep penetration, and the subsequent ROS production ensures that bacteria throughout the biofilm are killed and the matrix is dismantled. This dual action is a powerful feature that distinguishes many well‐designed CDs from simpler antimicrobial agents.

A more sophisticated and potentially transformative mechanism involves disrupting the regulatory networks that govern biofilm formation, rather than simply killing the constituent cells. By targeting QS and the expression of biofilm‐specific genes, CDs can “disarm” bacteria, rendering them unable to form a biofilm in the first place or to maintain an established one, without necessarily exerting strong selective pressure for resistance. Microbial biofilm formation is heavily dependent on cell‐to‐cell signaling mediated by QS molecules (Zhang, Bai, et al. 2023). Several studies have provided evidence that CDs can interfere with these pathways. Sadaqat et al. demonstrated that curcumin‐derived CDs (Cur‐CDs) downregulated the expression of *esp* (enterococcal surface protein) and *gelE* (gelatinase) in *Enterococcus faecium*, genes critical for adhesion and biofilm maturation (Sadaqat et al. 2022). Similarly, Liang et al. showed that tinidazole‐functionalized CDs reduced the mRNA expression of multiple biofilm‐related genes in *Porphyromonas gingivalis*, including *fimA* (long fimbriae A), *rgpA*, *rgpB*, and *kgp* (arginine‐ and lysine‐specific cysteine proteases) (Liang et al. 2020). These genes are essential for fimbriae production, protein processing, and adhesion to host tissues. By downregulating them, the CDs effectively inhibited the self‐assembly of the biofilm matrix (Liang et al. 2020). In the same study, the nanostructures themselves were proposed to enhance penetration and affect the production of fibrin, a key component of mature *P. gingivalis* biofilms (Liang et al. 2020). Lin et al. provided another compelling example, showing that bacteria‐derived CDs inhibited *E. coli* biofilm formation without affecting planktonic cell growth (Lin et al. 2018). This clear separation of anti‐biofilm and antibacterial activity strongly suggests a specific interference with regulatory pathways, such as sulfur metabolism or cyclic di‐GMP signaling (Lin et al. 2018; Wood 2009; Lee et al. 2007). This approach of “disarming” rather than killing represents a promising avenue for developing therapies that are less likely to drive the evolution of resistance.

The field is rapidly evolving beyond single‐mechanism designs toward sophisticated systems that combine multiple modes of action for enhanced efficacy. One promising approach is the conjugation of CDs with bioactive enzymes. Zhao et al. developed a CD‐LZM complex by coupling otherwise ineffective 4‐ACDs with lysozyme (Zhao et al. 2023). While the CDs provided penetration and delivery, the LZM actively hydrolyzed the peptidoglycan components of the *S. aureus* biofilm matrix, resulting in 86% inhibition at 60 μg/mL (Zhao et al. 2023). However, this complex inherited LZM's Gram‐positive specificity, highlighting both the potential and the limitation of enzyme‐conjugate strategies. Another innovative direction is photothermal therapy (PTT). Mauro et al. embedded CDs in a poly‐l‐lactic acid (PLA) scaffold, creating a material that could convert near‐infrared (NIR) light into localized heat (Mauro et al. 2020). This physical mechanism leads to rapid destabilization of the biofilm extracellular matrix and thermal killing of embedded cells, regardless of their species or drug resistance profile. The key advantage of PTT is that its physical mode of action is unlikely to induce resistance, making it ideal for self‐sterilizing medical devices and implants.

Targeted delivery represents a third emerging strategy. Yu et al. functionalized FA‐CDs, enabling them to bind specifically to folate receptors overexpressed on bacterial biofilms (Yu et al. 2022, 2021). This targeting enriches the CDs at the infection site, concentrating their therapeutic effect and potentially minimizing damage to surrounding healthy tissue. Finally, complex nanocomposites exploit synergistic effects from multiple components. Elkodous et al.‘s Co_x_Ni_1_ − xFe_2_O_4_/SiO_2_/TiO_2_/C‐dots system combines the photocatalytic activity of TiO_2_, the magnetic properties of the ferrite core, and the bioactivity of CDs (Abd Elkodous et al. 2020). The presence of Ti, Si, and C atoms on the nanocomposite surface was proposed to enhance penetration into pathogens, leading to the destruction of biological molecules (Abd Elkodous et al. 2020).

The antibiofilm activity of CDs is a complex, multidimensional phenomenon. The most effective systems are those that combine multiple mechanisms (such as electrostatic binding, deep penetration, ROS generation, and specific targeting) in a single nanoplatform. However, despite this progress, the field faces significant challenges. The lack of standardized testing protocols makes direct comparison across studies difficult. More importantly, the precise molecular events underlying many of these mechanisms remain poorly understood, particularly how CDs traverse the formidable Gram‐negative outer membrane and how they achieve selective toxicity against bacterial versus mammalian cells. Addressing these fundamental questions through detailed mechanistic studies will be essential for the rational design of next‐generation CDs with enhanced potency, specificity, and translational potential.

## 7 Conclusion and Future Perspectives

Interestingly, CDs have emerged as a promising class of carbon‐based nanomaterials with considerable potential for combating biofilm‐associated infections due to their unique physicochemical properties, including tunable surface chemistry, excellent aqueous dispersibility, intrinsic photoluminescence, ease of functionalization, and favorable biocompatibility. This review has comprehensively discussed the recent advances in CD synthesis strategies, physicochemical characterization, and the mechanisms underlying their antibiofilm activity. Both top‐down and bottom‐up synthesis methods have been highlighted, emphasizing how precursor selection, synthetic conditions, and surface functionalization significantly influence the structural characteristics and biological performance of CDs. Furthermore, the diverse antibiofilm mechanisms of CDs, including bacterial membrane disruption, ROS generation, EPS degradation, QS interference, inhibition of bacterial adhesion, and enhanced antimicrobial drug delivery, demonstrate their versatility as multifunctional nanoplatforms for preventing and eradicating microbial biofilms. Despite these encouraging developments, several challenges must be addressed before CDs can be translated into clinical and industrial applications. One of the primary limitations is the lack of standardized synthesis, purification, and characterization protocols, which often results in variability in particle size, surface functional groups, optical properties, and antibiofilm efficacy. Such inconsistencies make it difficult to compare findings across studies and hinder reproducibility. In addition, the molecular mechanisms governing the interactions between CDs and biofilm components remain incompletely understood, particularly regarding the influence of surface chemistry, heteroatom doping, particle size, and charge on antibacterial and antibiofilm performance. Although numerous studies have demonstrated promising in vitro results, relatively few have evaluated the long‐term biocompatibility, biodistribution, pharmacokinetics, biodegradation, immunogenicity, and environmental safety of CDs in vivo. Furthermore, challenges associated with large‐scale production, batch‐to‐batch consistency, long‐term storage stability, and regulatory approval continue to limit their practical implementation.

Several important research gaps remain to be explored. Future studies should establish standardized protocols for CD synthesis and physicochemical characterization to improve reproducibility and facilitate meaningful comparisons among different investigations. Advanced analytical and computational approaches should be employed to elucidate the structure–activity relationships that govern the antibiofilm properties of CDs. In addition, greater emphasis should be placed on evaluating their efficacy against clinically relevant multispecies biofilms and drug‐resistant pathogens using realistic infection models. Comprehensive in vivo investigations, followed by well‐designed preclinical and clinical studies, are essential to validate the safety and therapeutic efficacy of CD‐based antibiofilm systems. The integration of CDs with antibiotics, antimicrobial peptides, enzymes, hydrogels, photothermal and photodynamic therapies, as well as targeted drug delivery platforms, represents a promising strategy for enhancing therapeutic outcomes while minimizing antimicrobial resistance. Moreover, environmentally friendly and scalable green synthesis approaches, combined with artificial intelligence‐assisted nanomaterial design and regulatory standardization, may accelerate the development of next‐generation CD‐based antibiofilm technologies. The continuous advancement of synthesis methodologies, mechanistic understanding, multifunctional engineering, and translational research is expected to further expand the biomedical potential of CDs. Through interdisciplinary collaboration among materials scientists, microbiologists, chemists, engineers, and clinicians, CDs are well positioned to evolve from experimental nanomaterials into safe, effective, and clinically relevant platforms for the prevention, diagnosis, and treatment of biofilm‐associated infections.

## Author Contributions

**Hadeer M. Bedair:** conceptualization, methodology, project administration, writing – original draft, writing – review and editing. **Mahmoud Hamed:** conceptualization, investigation, writing – review and editing, data curation. **Aly A. Shoun:** investigation, conceptualization, writing – original draft, writing – review and editing, visualization, data curation. **Fotouh R. Mansour:** conceptualization, writing – original draft, writing – review and editing, project administration, methodology. **Reem H. Obaydo:** methodology, writing – review and editing, conceptualization, investigation, formal analysis. **Tamer M. Samir:** supervision, methodology, writing – original draft, writing – review and editing, project administration, validation.

## Funding

The authors have nothing to report.

## Ethics Statement

The authors have nothing to report.

## Conflicts of Interest

None declared.

## Data Availability

All data presented in the paper.
